# TDP-43 self-interaction is modulated by redox-active compounds Auranofin, Chelerythrine and Riluzole

**DOI:** 10.1038/s41598-018-20565-0

**Published:** 2018-02-02

**Authors:** Moritz Oberstadt, Jens Stieler, David Larbi Simpong, Ute Römuß, Nicole Urban, Michael Schaefer, Thomas Arendt, Max Holzer

**Affiliations:** 1 Department of Neurology, University of Leipzig, Liebigstraße 20, 04103 Leipzig, Germany; 2 Department for Molecular and Cellular Mechanisms of Neurodegeneration, Paul Flechsig Institute for Brain Research, University of Leipzig, Liebigstraße 19, 04103 Leipzig, Germany; 3 Rudolf-Boehm-Institute of Pharmacology and Toxicology, University of Leipzig, Härtelstraße 16-18, 04107 Leipzig, Germany

## Abstract

Amyotrophic lateral sclerosis (ALS) represents a fatal neurodegenerative disease, which is characterized by a rapid loss of lower and upper motor neurons. As a major neuropathological hallmark, protein aggregates containing the Transactivating Response Region (TAR) DNA Binding Protein (TDP-43) are detectable in about 95% of sporadic ALS patients. TDP-43 interacts with itself physiologically to form liquid droplets, which may progress to pathological aggregates. In this study, we established the NanoBit luciferase complementation assay to measure TDP-43 self-interaction and found the fusion of the split luciferase subunits to the N-terminus of the protein as the strongest interacting partners. A screen of pharmacologically active compounds from the LOPAC^®1280^ library identified auranofin, chelerythrine and riluzole as dose-dependent inhibitors of TDP-43 self-interaction. Further analysis of drug action of the gold-containing thioredoxin reductase inhibitor auranofin revealed a redistribution from insoluble TDP-43 protein pool to PBS-soluble protein pool in N2a cells. In addition, auranofin treatment diminished reduced glutathione as a sign for oxidative modulation.

## Introduction

Amyotrophic lateral sclerosis (ALS) represents a rapidly progressing neurodegenerative disease and is characterized by a degeneration of motor neurons in the motor cortex and the spinal cord. Clinical symptoms include weakness of muscles and spasticity which may result in loss of ambulation and of arm and hand function, difficulty with speech and swallowing and finally a failure of respiratory musculature^1^. Frequently, motor deficits arise from one particular region and appear to spread to contiguous anatomic regions. According to one hypothesis, the pathogenesis involves prion-like transsynaptic propagation of an abnormal protein from one to the neighboring cortical or spinal motor neuron^2,3^. As a neuropathological hallmark of ALS, protein aggregates have been found in motor neurons of ALS patients which contain a variety of proteins like profilin 1 or peripherin involved in different cellular functions like the intracellular transport or the cytoskeleton architecture^4,5^. The Transactivating Response Region (TAR) DNA Binding Protein (TDP-43) is a major component of these cytoplasmic protein aggregates and detectable in about 95% of sporadic ALS patients^6,7^. TDP-43 is a multidomain protein containing a folded, multimer-forming N-terminal domain^8,9^, tandem RNA recognition motif (RRM) domains that bind (UG)-rich sequences^10^, and a C-terminal domain (CTD) that is essential for heterogeneous ribonucleoprotein particle (hnRNP) interactions and splicing activity^11^. In physiological context, TDP-43 is localized in the nucleus and involved in regulation of RNA transcription^12^. During stress response, TDP-43 is translocated into the cytoplasm and participates in stress granule assembly, forming a RNA rich cytoplasmic subcompartment with liquid droplet-like characteristics. In ALS, cytoplasmic TDP-43 aggregates are thought to contribute to neurodegeneration of motor neurons^13^. TDP-43 physically interacts with itself forming dynamic aggregates together with stress granule proteins Fus, TIA1 and G3BP^14,15^. Several protein regions of TDP-43 like N-terminus, RNA recognition motif (RRM) domains and C-terminus are involved in the aggregation process^11,16,17^.

TDP-43 assemblies may represent a crucial intermediate state prior to TDP-43 aggregation^18^. Therefore, substances modulating TDP-43 self-interaction may interfere with formation of pathogenic oligomeric aggregates and hold therapeutic promise. For measurement of TDP-43 self-interaction, NanoBit luciferase complementation assay has been applied^19^. Two different NanoLuc luciferase subunits (large Bit (lgBit), an 18 kDa polypeptide, and small bit (smBit), a 1.3 kDa peptide) have been fused to the N-terminal and/or C-terminal site of TDP-43 protein, respectively. The NanoBit fusion-subunits only weakly associate by themselves (K_D_ > 100 µM), so that their reconstitution of luciferase activity is controlled by the interaction characteristics of the tagged proteins^19,20^.

In our study, we found the highest self-interaction potential of constructs pFN33_TDP-43 and pFN35_TDP-43 with each of the NanoBit subunits fused to the N-terminal site of TDP-43 having a parallel TDP-43 orientation and allowing an undisturbed self-interaction of other regions like RRM domains or C–terminus of TDP-43. Testing pharmacologically active compounds from LOPAC^®1280^ library, we found riluzole, auranofin and chelerythrine as substances, which significantly decreased TDP-43 self-interaction. Especially auranofin showed impressive results with a straight, dose-dependent reduction of TDP-43 self-interaction without cell toxicity effects. Furthermore, auranofin seems to improve TDP-43 solubility as exemplified by a redistribution of TDP-43 from Triton X-100 and urea-soluble protein pool to PBS-soluble protein pool.

## Results

### TDP-43 self-interaction

**Figure 1:**
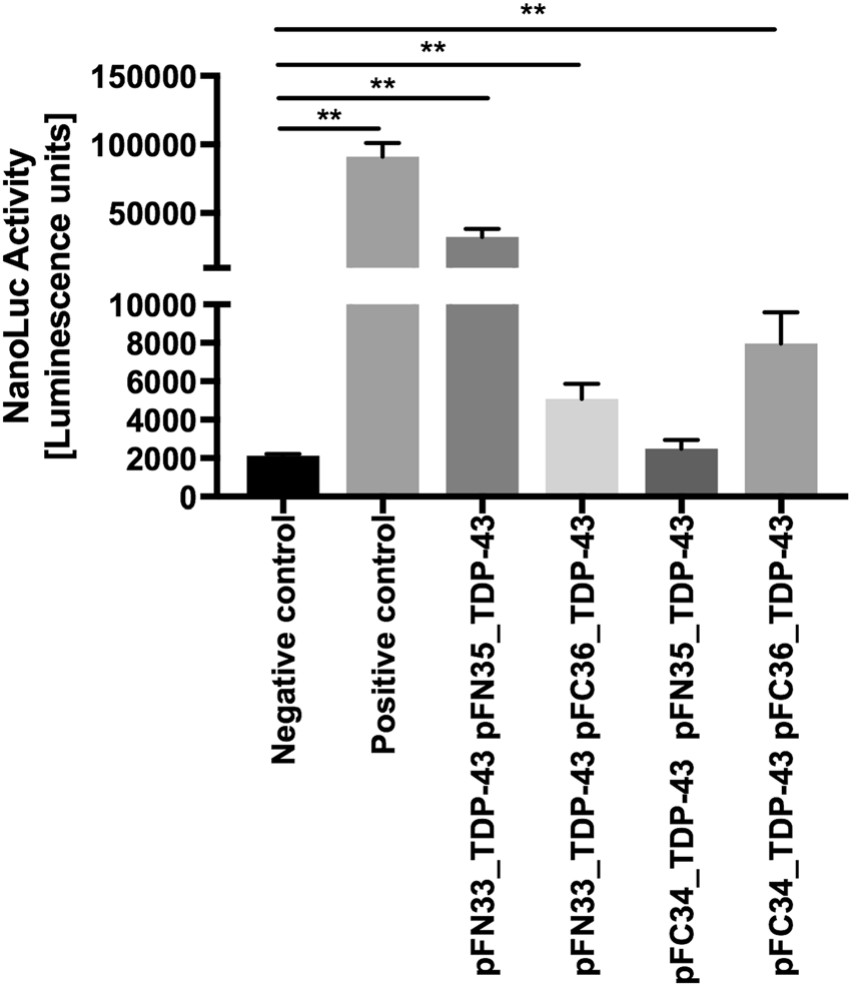
TDP-43 self-interaction. NanoBit^®^ luciferase complementation assay for protein interactions has been used to measure interaction of different N-terminal large (LgBit, pFN33), small (SmBit, pFN35), C-terminal large (LgBit, pFC34) and small (SmBit, pFC36) fusion proteins of TDP-43, positive control vectors SmBiT-PRKACA and LgBiT-PRKAR2A (coding the catalytic and regulatory subunits of PKA) and vector SmBiT-Halotag, which contains haloalkane dehalogenase – smBit fusion protein (Halitag) as a negative control, were obtained from Promega (Madison, WI, USA) and have been used in parallel 24 h after transfection of N2a cells and seeding cells into 384-well plates, Mean ± SD, Mann-Whitney-U-Test **p < 0.01.

For evaluation of the best configuration we tested four different pairings of TDP-43 fusion proteins. Fusion of both the large bit and the small bit to the N-Terminus of TDP-43 resulted in the highest luciferase activity (Fig. 1). Second best was the pairing where both luciferase bits were fused to the C-terminus to TDP-43, pointing to a parallel interaction of TDP-43. Antiparallel orientation of the luciferase bits yielded luciferase activity barely higher than the negative control (Fig. 1, first column).

### Screening of pharmacologically active compounds from LOPAC^®1280^ library on TDP-43 self-interaction (single point determination)

**Figure 2:**
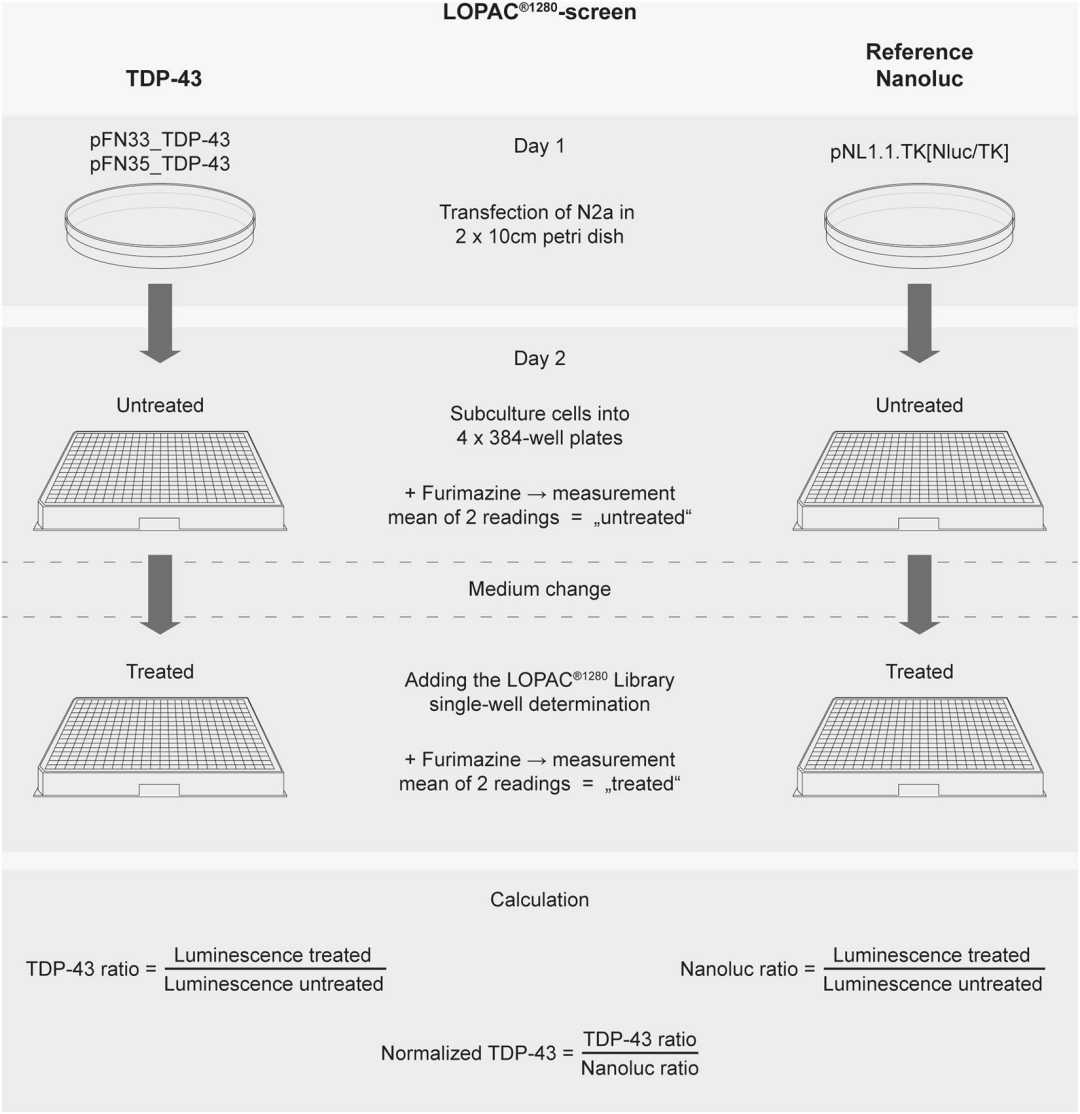
Flowchart of NanoBit luciferase measurement with LOPAC^®1280^-screen. Description of measurement procedure with compounds from LOPAC^®1280^ library. For reduction of variability, luciferase reading from each well after treatment has been normalized to the same well before treatment. To detect off-target effects of compounds such as interference with luciferase reaction, protein homeostasis and TK promoter activity, we applied the same compounds onto N2a cells transfected with a TK-promoter construct expressing full length NanoLuc luciferase. Therefore, results of TDP-43 self-interaction were normalized with respect to NanoLuc activity after library treatment.

LOPAC^®1280^ library, a collection of inhibitors, receptor ligands, pharma-developed compounds and approved drugs covering most signaling pathways and major drug target classes was applied at 10 µM concentration to the transfected mouse neuroblastoma Neuro2a (N2a) cells for the TDP-43 self-interaction assay. Screening the library compounds was performed as single determination in 384 wells. In order to reduce variability, we obtained a luciferase reading from each well after treatment and normalized to the luciferase reading of the same well before treatment (Fig. 2). Further, to detect off-target effects of compounds such as interference with luciferase reaction, protein homeostasis and TK promoter activity, we applied the same compound library onto N2a cells transfected with construct expressing full length constitutively-active NanoLuc luciferase driven by the same thymidine kinase promoter like the TDP-43 construct. Therefore, results of TDP-43 self-interaction were normalized with respect to NanoLuc activity after library treatment (Fig. 2). Compounds causing a reduction of TDP-43 interaction self-interaction to 70% [after%/pre%] or lower were considered as inhibitors and compounds, which increased the interaction to 120% [after%/pre%] or higher have been considered as stimulators (Table 1).

**Table 1:** Effects of selected pharmacologically active compounds from LOPAC®1280 library on TDP-43 self-interaction. Interaction of constructs pFN33_TDP-43 and pFN35_TDP-43 under treatment with compounds of the LOPAC®1280 library, a collection of inhibitors, receptor ligands, pharma-developed tools, and approved drugs covering most signaling pathways and all major drug target classes. Data have been referred to N2a cells transfected with Nluc construct as control. Compounds, which changed the TDP-43 self-interaction to 70% [after%/pre%] or lower have been considered as inhibitors and compounds, which increased the interaction to 120% [after%/pre%] or higher have been considered as stimulators. Chosen compounds for further investigations are written in italic.

TDP-43 [after%/pre%]	Name	Class	Action	Description
*Inhibition*				
30	(±)-AMT hydrochloride	Nitric oxide	Inhibitor	Nitric oxide synthase inhibitor
38	GABA	GABA	Agonist	Endogenous neurotransmitter
39	Sanguinarine chloride	Ion Pump	Inhibitor	Inhibitor of Mg^2+^ and Na + /K + -ATPase
61	I-OMe-Tyrphostin AG 538	Phosphorylation	Inhibitor	Insulin growth factor 1 (IGF-1) receptor protein tyrosine kinase inhibitor
*63*	***Auranofin***	***Phosphorylation***	***Inhibitor***	***Inhibitor of selenoenzyme thioredoxin reductase (TrxR)***
63	Ruthenium red	Ion Pump	Inhibitor	Inhibitor of mitochondrial Ca^2+^ uniporter
*64*	***Chelerythrine chloride***	***Phosphorylation***	***Inhibitor***	***PKC inhibitor***
64	p-Aminoclonidine hydrochloride	Adrenoceptor	Agonist	Alpha2 Adrenoceptor agonist
65	L-allylglyciner	Biochemistry	Inhibitor	Glutamic acid decarboxylase inhibitor
66	1-Aminocyclopropanecarboxylic acid hydrochloride	Glutamate	Agonist	NMDA glutamate receptor agonist acting at the glycine site
67	Fluvoxamine maleate	Antidepressant	Inhibitor	Selective serotonin reuptake inhibitor
*69*	***Oxotremorine sesquifumarate salt***	***Cholinergic***	***Agonist***	***Muscarinic acetylcholine receptor agonist with preference for the M2 receptor***
70	S-Nitrosoglutathione	Nitric Oxide	Donor	Nitric oxide donor
70	9-Amino-1,2–3,4-tetrahydroacridine hydrochloride	Cholinergic	Inhibitor	Cholinesterase inhibitor
70	THIP hydrochloride	GABA	Agonist	GABA-A receptor agonist
*82*	***Riluzole***	***Glutamate***	***Antagonist***	***Glutamate release inhibitor***
*Stimulation*				
120	VER-3323 hemifumarate salt	Serotonin	Agonist	5-HT2C/5-HT2B serotonin receptor agonist
125	Vancomycin hydrochloride from Streptomyces orientalis	Antibiotic	-	Amphoteric glycopeptide antibiotic
126	Ritodrine hydrochloride	Adrenoceptor	Agonist	Beta2-Adrenoceptor agonist
126	CGP-7930	GABA	Modulator	Positive allosteric modulator of GABA-B receptors
126	R( + )-Butylindazone	Ion Pump	Inhibitor	[K + , Cl-]-cotransport inhibitor
127	GR-89696 fumarate	Opioid	Agonist	Kappa Opioid receptor agonist
128	Cefsulodin sodium salt hydrate	Antibiotic	-	Third generation cephalosporin antibiotic
131	S15535	Serotonin	Agonist	Partial 5-HT1A receptor agonist
141	Indirubin-3’-oxime	Phosphorylation	Inhibitor	Cyclin-dependent kinase inhibitor
*146*	***Suramin sodium salt***	***P2 Receptor***	***Antagonist***	***P2X and P2Y receptor antagonist***
*150*	***Reactive Blue 2***	***P2 Receptor***	***Antagonist***	***P2Y receptor antagonist***
164	N-Bromoacetamide	Na^+^ Channel	Modulator	Chemical modifier
230	1-Amino-1-cyclohexanecarboxylic acid hydrochloride	Neurotransmission	Substrate	Synthetic amino acid that crosses the blood-brain barrier by the Large Neutral Amino Acid carrier system

**Table 2:** Effects of GABA, Reactive Blue 2, Suramin sodium salt, Riluzole, Auranofin, Oxotremorine sesquifumarate salt and Chelerythrine on TDP-43 self-interaction. Interaction of constructs pFN33_TDP-43 and pFN35_TDP-43 under treatment with GABA, Reactive Blue 2, Suramin sodium salt, Riluzole, Auranofin, Oxotremorine sesquifumarate salt and Chelerythrine. Results described in % of untreated reference for each compound concentration (1.6–62.5 µM) in TDP-43 NanoBit luminescence, Mean ± SD. Results for compound concentration 10.0 µM are written in bold, because this concentration has been previously used in LOPAC®1280 library screen.

Substance	Concentration	% of Reference
**GABA**	1.6 µM	127.5 ± 33.6
	4.0 µM	114.3 ± 28.5
	**10.0 µM**	**112.4** ± **23.7**
	25 µM	105.3 ± 27.6
	62.5 µM	112.9 ± 33.4
**Reactive Blue 2**	1.6 µM	90.7 ± 17.0
	4.0 µM	111.4 ± 6.5
	**10.0 µM**	**86.4 ± 18.8**
	25 µM	92.6 ± 17.7
	62.5 µM	88.3 ± 28.2
**Suramin sodium salt**	1.6 µM	200.1 ± 9.9
	4.0 µM	106.7 ± 14.3
	**10.0 µM**	**99.6 ± 38.6**
	25 µM	97.8 ± 17.9
	62.5 µM	110.0 ± 5.3
**Riluzole**	1.6 µM	118.7 ± 13.1
	4.0 µM	68.9 ± 16.4
	**10.0 µM**	**82.9 ± 21.4**
	25 µM	58.2 ± 10.0
	62.5 µM	27.9 ± 5.3
**Auranofin**	1.6 µM	74.5 ± 12.8
	4.0 µM	28.7 ± 24.2
	**10.0 µM**	**8.8 ± 4.1**
	25 µM	0.9 ± 2.5
	62.5 µM	0.5 ± 3.6
**Oxotremorine sesquifumarate salt**	1.6 µM	195.0 ± 10.4
	4.0 µM	117.1 ± 12.7
	**10.0 µM**	**112.9 ± 14.1**
	25 µM	123.2 ± 24.0
	62.5 µM	103.9 ± 23.4
**Chelerythrine**	1.6 µM	83.1 ± 20.9
	4.0 µM	61.4 ± 10.8
	**10.0 µM**	**23.7 ± 9.6**
	25 µM	7.6 ± 2.7
	62.5 µM	9.1 ± 6.3

From the list of potential hits, we selected seven compounds for validation and for obtaining a dose-response curve. We specifically tested the following compounds GABA (38% of untreated cells), Auranofin (63% of untreated cells), Chelerythrine chloride (64% of untreated cells), Oxotremorine sesquifumarate salt (69% of untreated cells), Riluzole (82% of untreated cells), Suramin sodium salt (146% of untreated cells) and Reactive Blue 2 (150% of untreated cells).

### Dose-response relationship for selected compounds

**Figure 3:**
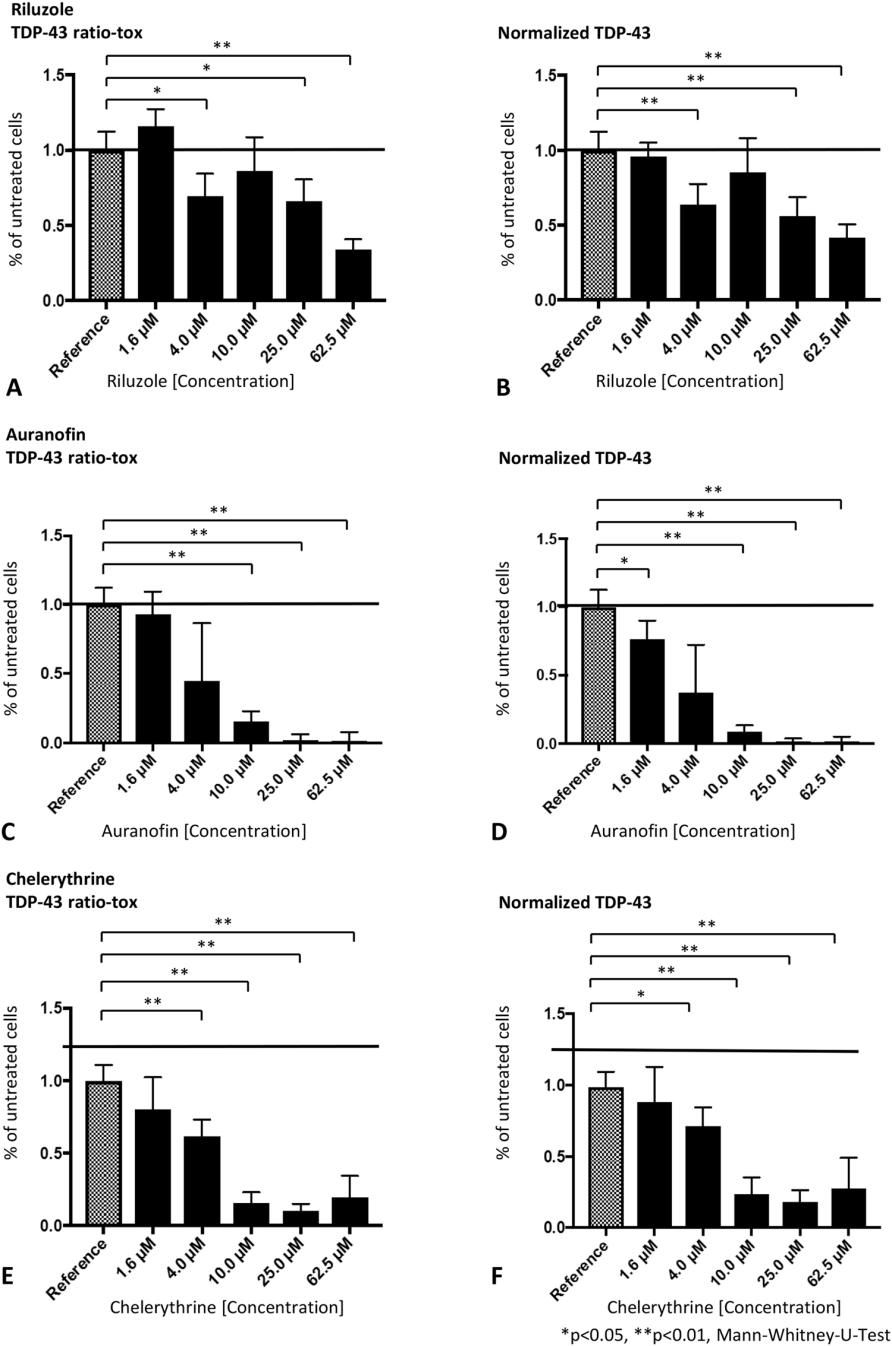
TDP-43 self-interaction under treatment with compounds riluzole, auranofin and chelerythrine. Interaction of constructs pFN33_TDP-43 and pFN35_TDP-43 under treatment with compounds riluzole (**A** + **B**), auranofin (**C** + **D**) and chelerythrine (**E** + **F**) measured with established NanoBit complementation reporter assay. Results described in % of untreated reference for each compound concentration (1.6–62.5 µM), initially in TDP-43 NanoBit luminescence in relation to cell proliferation WST-8 assay (**A**,**C** + **E**) and as TDP-43 NanoBit luminescence compared to NanoLuc control NanoBit luminescence (**B**,**D** + **F**) for each treatment, Mean ± SD, Mann-Whitney-U-Test, *p < 0.05, **p < 0.01, n = 6.

**Figure 4:**
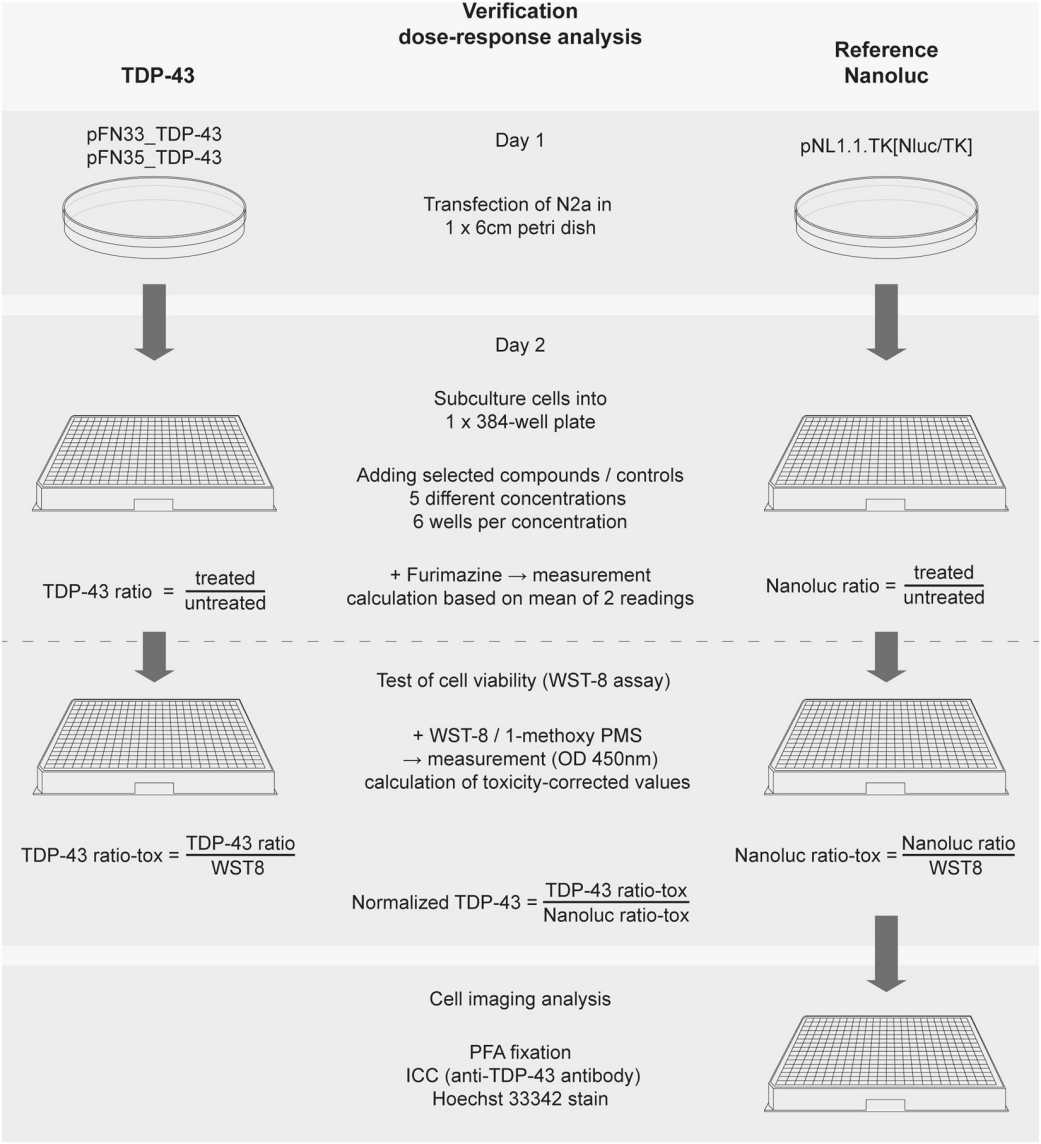
Flowchart of NanoBit luciferase measurement for verification dose-response analysis. Description of measurement procedure for selected compounds from LOPAC^®1280^ library. For reduction of variability, luciferase reading from each well after treatment has been normalized to the same well before treatment (TDP-43 ratio). To detect effects on cell viability, TDP-43 ratio has been calculated in relation to cell viability measurement via WST-8 assay in the same well (TDP-43 ratio-tox). For avoidance of off-target effects of compounds such as interference with luciferase reaction, protein homeostasis and TK promoter activity, we applied the same compounds onto N2a cells transfected with a TK-promoter construct expressing full length NanoLuc luciferase. Therefore, results of TDP-43 self-interaction were normalized with respect to NanoLuc activity (Normalized TDP-43).

In our validation study we observed a dose-dependent reduction of TDP-43 self-interaction for the substances riluzole, auranofin and chelerythrine (Fig. 3), while effects of the substances GABA, Oxotremorine sesquifumarate salt, Suramin sodium salt and Reactive Blue 2 have not been reproducible (Table 2). For the dose-response experiments we applied the compounds at final concentrations of 1.6 µM, 4.0 µM, 10.0 µM, 25.0 µM and 62.5 µM to both the TDP-43 interaction assay and the NanoLuc reference assay (Fig. 4). The WST-8 assay has been measured in the same wells for evaluation of cell viability immediately after luciferase readings (Supplemental Fig. 2). Riluzole treatment resulted in a significant reduction of TDP-43 interaction at concentrations of 4 µM (64% of untreated cells), 25 µM (56%) and 62.5 µM (42%), while TDP-43 interaction at concentration of 10 µM did not show a significant reduction explainable by experimental variability within the 6-fold replicates (85%; Fig. 3A). After normalization with luciferase readings from riluzole treated cells expressing the constitutively active NanoLuc luciferase, the calculated reduction for 4 µM, 25 µM and 62.5 µM riluzole amounted to 64%, 56% and 42% of untreated cells, respectively (Fig. 3B).

**Table 3:** TDP-43 primers.

Primer name	Sequence
TDP-43-R	5′-GGAGACCCAACACTATTAAATCGG-3′
TDP-43-CTF	5′-GCAGAGGGAGCCAAACCAGG-3′
Nano-R	5′-GTTAGCAGCCGGATCAGCTTG-3′

Auranofin treatment at a concentration of 4 µM caused a non-significant reduction of TDP-43 self-interaction (45% of non-treated cells), whereas auranofin concentrations of 10 µM (15.4%), 25 µM (1.5%) and 62.5 µM (1.1%) had a strong and significant effect (Fig. 3C). After normalization of the TDP-43 interaction results to the constitutive NanoLuc, effects became more evident revealing that even the lowest concentration of 1.6 µM auranofin resulted in a significant reduction of 76% of non-treated cells (Fig. 3D).

Chelerythrine treatment at a concentration of 4 µM induced a significant reduction in TDP-43 interaction to 62%. Higher chelerythrine concentrations had much stronger effects 10 µM (15.6% of non-treated cells), 25 µM (10%), 62.5 µM (19.5%; Fig. 3E). After normalization with luciferase readings from chelerythrine-treated cells expressing the constitutively active NanoLuc luciferase (Fig. 3F), effects on TDP-43 were less pronounced at 4 µM (71% of non-treated cells), 10 µM (24%), 25 µM (18%) and 62.5 µM chelerythrine (28%) owing to a possible toxic effect to the N2a neuroblastoma cells at the highest concentration during the 60 min incubation. In summary, the validation experiments for these three compounds riluzole, auranofin and chelerythrine showed a dose-dependent reduction of TDP-43 self-interaction with significant results at 4 µM to 10 µM concentration. Auranofin treatment resulted in the strongest, dose-dependent reduction of TDP-43 self-interaction without exerting cell toxicity in the observation time-frame.

### Endogenous TDP-43 expression under treatment with riluzole, auranofin and chelerythrine

**Figure 5:**
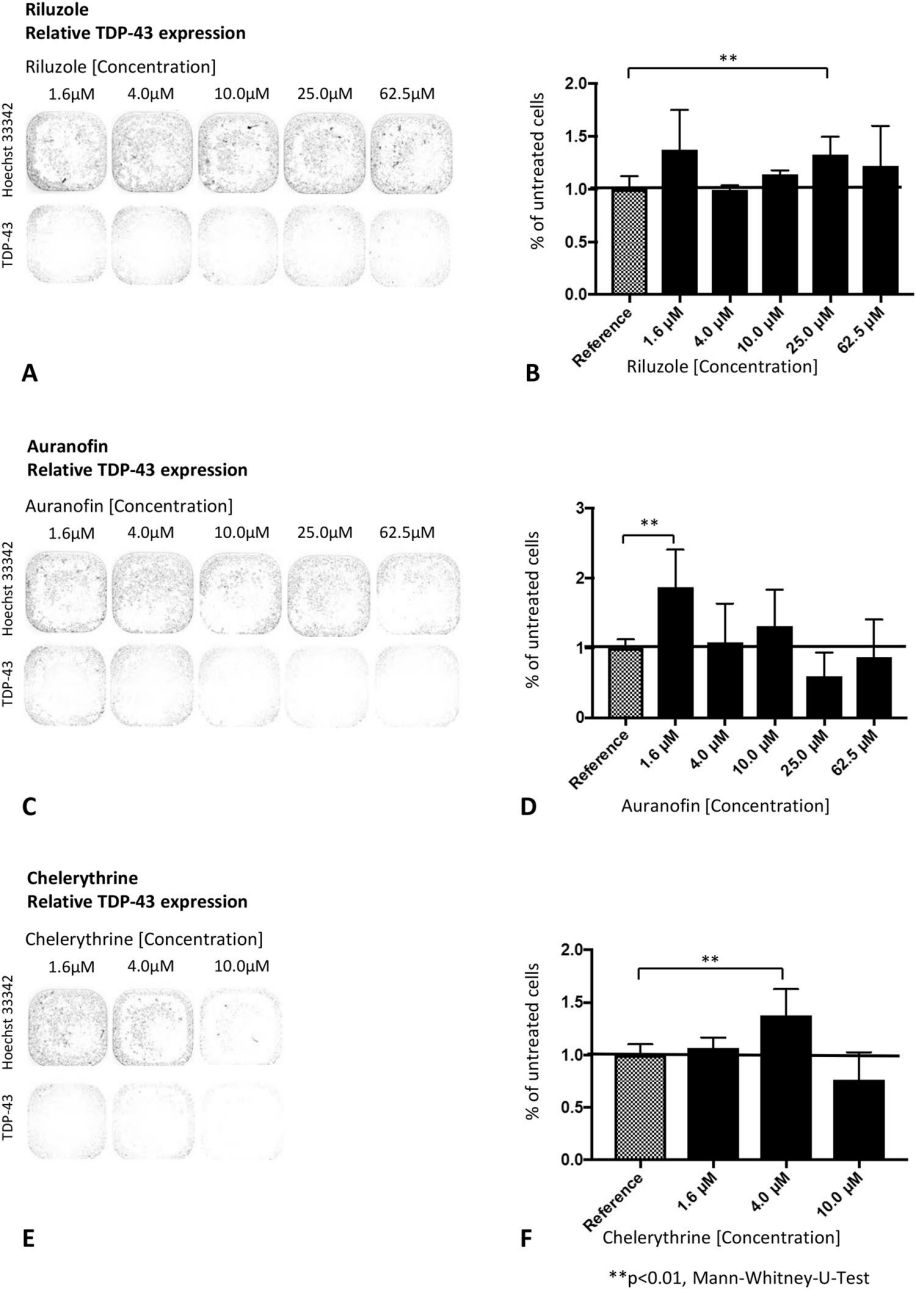
Relative TDP-43 expression under treatment with compounds riluzole, auranofin and chelerythrine. N2a cells fixed with 4% paraformaldehyde after treatment with riluzole (**A** + **B**), auranofin (**C** + **D**) and chelerythrine (**E** + **F**) in different concentrations (1.6–62.5 µM) and stained with anti-TDP-43 and DAPI. Results described in % of untreated reference for each compound concentration (1.6–62.5 µM; **B**,**D** + **F**), Mean ± SD, Mann-Whitney-U-Test, **p < 0.01, n = 6.

Because the cellular amount of the LgBit- and SmBit-TDP-43 fusion protein is much lower than the endogenous TDP-43 content (Supplemental Fig. 3), we assume that endogenous TDP-43 present in N2a cells can affect the read-out of the NanoBit interaction assay by competing with the Plasmid-coded TDP-43 fusion proteins. In order to estimate a possible impact of the compounds on the steady-state level of endogenous TDP-43 protein, we performed an immunocytochemistry-based quantification. NanoLuc-only transfected N2a cells of the verification experiments have been fixed and permeabilized after luciferase and WST-8 measurement, and labeled with anti-TDP-43 antibody and Hoechst 33342 (Fig. 5). This procedure allowed us to estimate changes in nuclear TDP-43 by fluorescence densitometry after pharmacological treatment in Hoechst 33342 positive nuclei (Fig. 5A,C,E). We observed an increase in endogenous TDP-43 at low compound concentrations such as 1.6 µM riluzole (37% increase, non-significant), 1.6 µM auranofin (88% increase, p < 0.01) and 4 µM chelerythrine (38%, p < 0.01, Fig. 5B,D,F). Higher concentrations of these three compounds (10 µM–62.5 µM) did not significantly change endogenous nuclear TDP-43 protein. In the presence of chelerythrine at concentrations of 25 µM and 62.5 µM, the number of cells was drastically reduced (Fig. 5E). Therefore, relative TDP-43 expression cannot be reliably established at these concentrations. Chelerythrine especially reduced number of Hoechst 33342 stained cell nuclei in higher concentrations (25 µM to 62.5 µM, Fig. 5E) – probably due to its cell toxicity, but did not show a dose-dependent effect on relative TDP-43 expression (Fig. 5F). Taken together, these results lead us to conclude that slightly changes in the TDP-43 steady state levels are unlikely to cause the reduction of TDP-43 self-interaction of treated N2a cells.

### Intracellular shift of TDP-43 from the particulated to the cytosolic cell fraction under auranofin treatment

**Figure 6:**
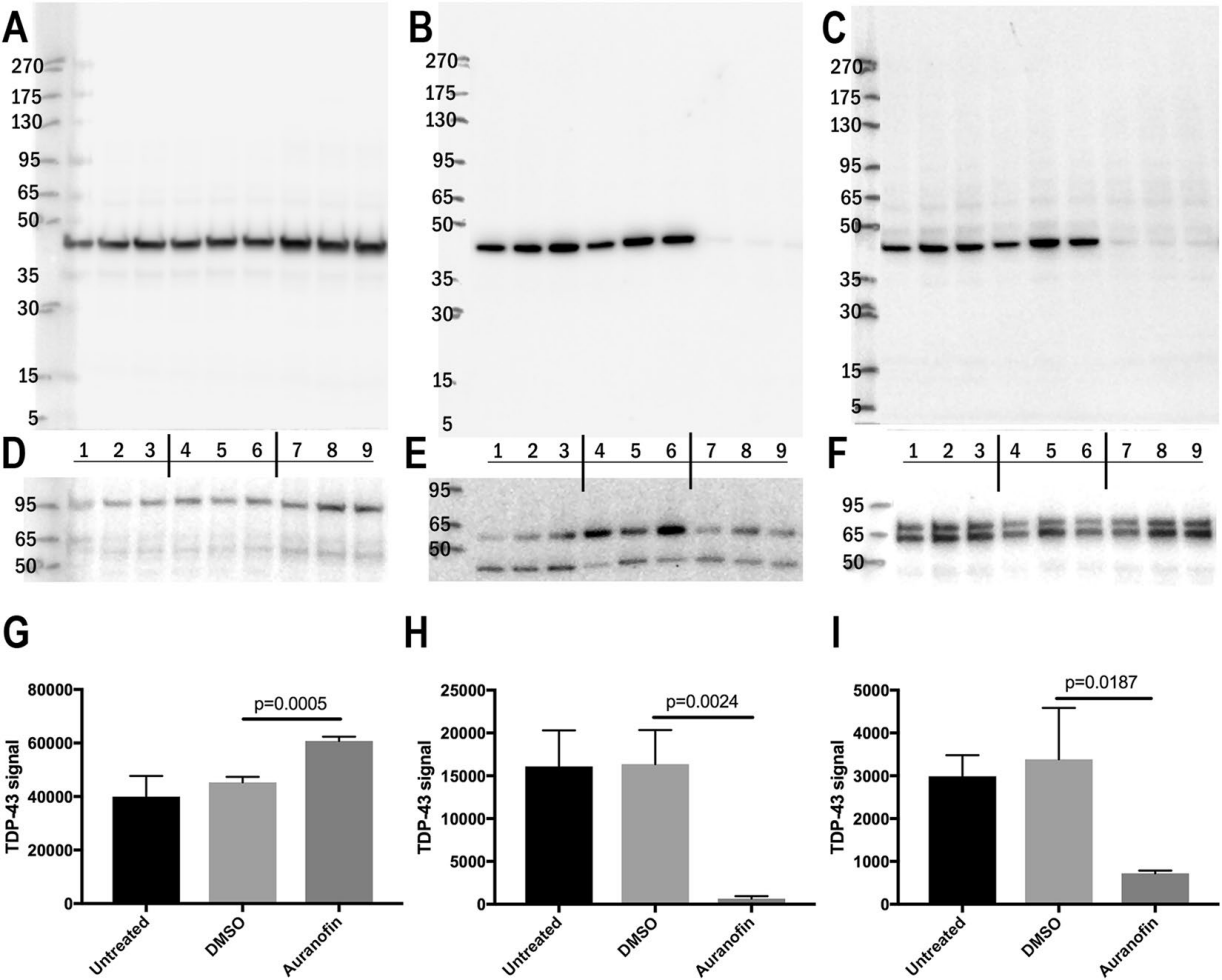
Sequential extraction of TDP-43 after auranofin treatment of N2a cells. Sequential extraction of TDP-43 has been performed of untreated N2a cells (lanes 1–3) and cells treated with 0.1% DMSO (lanes 4–6) as a vehicle control or 10 µM auranofin (lanes 7–9). Soluble proteins obtained after ultrasonication in PBS and ultracentrifugation have been probed for TDP-43 (**A**) and Hsp90 (**D**) as cytoplasmic reference protein. Luminal and membrane-associated proteins were extracted in 1% Triton X-100 containing PBS buffer, ultracentrifuged and probed for TDP-43 (**B**) and protein disulfide isomerase (**E**) as a luminal ER reference protein. Protein aggregates, cytoskeletal and nuclear proteins were solubilized by 8 M urea in PBS buffer and probed for TDP-43 (**C**) and Lamin A/C (**F**) as nuclear matrix protein. There is a strong reduction of TDP-43 extractable with Triton X-100 or 8 M urea after auranofin treatment (compare lanes 7–9 to lanes 4–6 **B**,**H** and **C**,**I**, respectively) whereas the PBS soluble fraction of auranofin treated N2a cells yields a 34% increase in TDP-43 signal (**A**,**G**). Signal intensities were quantified by densitometry on uncropped raw pictures (Supplementary Figure 6) using TINA 2.09 software (Raytest, Germany). Mean and standard deviation are depicted in **G**,**H**,**I**. Error probability was calculated with Student’s T-test, n = 3. For sake of better presentability contrast levels of different extractions were normalized, for uncropped raw data and full-length blots see Supplementary Figure 6.

To further analyze the effect of auranofin treatment on TDP-43 self-interaction in terms of cellular TDP-43 distribution we performed a sequential protein extraction of TDP-43 after auranofin treatment of N2a cells. Auranofin treatment resulted in a massive reduction of TDP-43 in the Triton X-100 soluble (Fig. 6B,H) and urea soluble fraction (Fig. 6C,I) compared to DMSO control (p = 0.0024 and p = 0.0187, respectively, Student’s t Test, Fig. 6). In parallel, the PBS-soluble fraction of TDP-43 increased by 34% (p = 0.0005, Fig. 6A,G). To further characterize the molecular weight of TDP-43 assemblies in the soluble fraction, we used size-exclusion chromatography on superose 6 column (separation range: 5 MDa −5 kDa) coupled to TDP-43 ELISA. We obtained two TDP-43 peaks at 8.3–9.8 ml as well as 16.8–18.3 ml (Supplemental Fig. 5), similar to TDP-43 distribution in soluble fractions in previous studies with TDP-43 detection at fractions 2–4 and 9–12 in one-milliliter column fractions^21^ and by Johnson *et al*. with an oligomer fraction at ca. 669 kDa and dimer and monomer peaks at 67 and 35 kDa, respectively^22^. Treatment with 10 µM auranofin did not change much the TDP-43 distribution in size-exclusion chromatography. There was a slight increase at 7 kDa TDP-43 peak. Thus, auranofin treatment results in an intracellular shift of TDP-43 to the soluble protein pool dissolving it from particle-bound interactions as shown by sequential protein extraction, but does not alter the association of TDP-43 with soluble protein assemblies in the cell.

### Redox effect of auranofin treatment

Because we aimed to find the potential drug mechanism, by which auranofin modulates TDP-43 self-interaction, we analyzed the redox effect of the known thioredoxin reductase inhibitor auranofin^23^ onto the cellular glutathione redox buffer (Supplemental Fig. 4). Treatment with Auranofin 10 µM (Mean fluorescence intensity (MFI): 1072) and 25 µM (MFI: 1210) resulted in a dose-dependent oxidative shift compared to DMSO baseline (MFI: 833). Thus, we detected a dose-dependent loss of reduced glutathione by auranofin in N2a cells.

## Discussion

TDP-43 is a major component of cytoplasmic proteinaceous aggregates and detectable in about 95% of sporadic ALS patients^6,7^. Thus, TDP-43 aggregation is seen as a neuropathological hallmark of ALS^4^. Beside ALS, TDP-43 inclusions have been observed in a broad spectrum of neurodegenerative disorders such as ALS/parkinonism-dementia complex of Guam, Alzheimer disease, dementia with Lewy bodies, Pick’s disease, argyrophilic grain disease and corticobasal degeneration^24^. The TDP-43 aggregation appears to be cooperatively mediated by several protein regions like N-terminus, RNA recognition motif (RRM) domains and C-terminus^11,16,17^.

In our study, we analyzed the TDP-43 protein-protein self-interaction using NanoBit complementation reporter assay, which allows sensitive detection of protein-protein interaction with low expression levels of exogenous split fusion proteins based on luminescence compared to bimolecular fluorescence complementation assay (BiFC) based on fluorescence^19,25^. The highest self-interaction potential has been observed in the constructs pFN33_TDP-43 and pFN35_TDP-43 with each of the NanoBit subunits fused to the N-terminal site of the protein allowing an undisturbed self-interaction of other regions like RRM domains or C–terminus of TDP-43. We tested modulation of TDP-43 self-interaction by pharmacologically active compounds from LOPAC^®^1280 library and found a reproducible, dose-dependent reduction of TDP-43 self-interaction by auranofin without reduction of endogenous TDP-43 expression and without cell-toxicity effects.

Beside some false hits from our initial screen we detected few substances which diminished TDP-43 self-interaction in our validation study such as Riluzol, auranofin, chelerythrine and sanguinarine (not shown). In our further analysis, we concentrated on auranofin, an organic gold thiol compound, because of its strong inhibition and good toxicity characteristics and due to its clinical use in therapy of rheumatoid arthritis to reduce inflammation^23,26^. It acts as a thioredoxin reductase inhibitor and competitively binds reduced selenocysteine located within the enzyme redox center^23^. Specifically, it is thought that the C-terminal redox-active Cys-495/SeCys-496 center of thioredoxin reductase is the target of auranofin^23,27^. Six cysteine residues have been also described in TDP-43 protein, four of which (Cys173, Cys175, Cys198 and Cys244) are located in the two RRM domains, while the other two (Cys39 and Cys50) are in the N-terminal domain^28^. Oxidation of cysteines located in the two RRMs decreases protein solubility, and leads to the formation of intra- and inter-molecular disulfide linkage^29,30^. Furthermore, cysteine residues in RRM1 may direct the conformation of TDP-43^31^. Thus, auranofin may be involved in regulation of TDP-43 self-interaction by modulating the cysteine residues in RRM domains of TDP-43. Beside this, auranofin induces a striking intracellular redistribution of TDP-43 from particulate protein fraction to the PBS-soluble protein fraction as shown by sequential protein extraction. This was obvious in Triton X-100 extractable particulate protein fraction, which may represent luminal and membrane-bound protein from cell membrane and organelles such as mitochondria and endosomes or even membrane-less liquid-like droples^32^, in which TDP-43 is enriched. In the subsequent 8 M urea extractable protein pool again TDP-43 was largely depleted after auranofin treatment. This protein pool comprises nuclear matrix protein, cytoskeletal proteins and protein complexes that resisted preceeding extractions. Thus, auranofin seems to release its inhibitory effect on TDP-43 self-interaction either by shifting it from membrane-bound organelles to the cytoplasm, by dissolving TDP-43 containing liquid-like droplets or by diminishing TDP-43 from subnuclear compartments. This finding is complemented by the size-exclusion chromatography results for PBS-soluble TDP-43, which shows that the molecular weight of the main TDP-43 peaks remain unchanged but that the 67 kDa peak is increased.

Interestingly, auranofin has been shown to reach CNS concentrations above 0.1 μM after oral administration in rats and mice^26^ suggesting that pharmacokinetic properties of auranofin may allow to explore its therapeutic efficacy in ALS. Further, because auranofin has redox-modulating properties, it would target an additional pathogenetically important mechanism as indicated by the disease-modifying effect of free-radical scavenger edaravone in some ALS patients^33^.

Beside auranofin, riluzole showed a significant inhibition of TDP-43 self-interaction without cell toxicity and without change in TDP-43 expression. At present, riluzole is the only drug in Europe approved for treatment of ALS based on its moderate increase of survival time by 3 months^34–36^. Mechanistically, riluzole is thought to have a broad spectrum of action with reduction of excitotoxicity by the excitatory amino acid neurotransmitter glutamate by enhancing glutamate uptake through glutamate transporters GLAST, GLT1 and EAAC1^35,37^, inhibition of voltage-dependent sodium channels^38^ and potassium channels^39^ and inhibition of protein kinase C, suggesting its involvement in antioxidative processes^40^. Further, oxidative modulation of cysteine residues during regulation auf K^+^ channels has been reported^41^. The present findings appear to be the first to demonstrate modulation of TDP-43 self-interaction suggesting that inhibition of TDP-43 self-interaction may be an additional mechanism by which riluzole may exert its beneficial effect in ALS. Because riluzole has antioxidative properties, it may inhibit TDP-43 aggregation by a mechanism similar to auranofin.

Finally, we found chelerythrine to be a strong inhibitor of TDP-43 self-interaction. While its application did not result in a reduced TDP-43 expression, substantial cell toxicity was found in concentrations of 10 µM and above. Chelerythrine is a benzophenanthridine alkaloid from the plant Chelidonium majus. It acts as a potent, selective and cell-permeable protein kinase C inhibitor^42^ and as antagonist of G-protein-coupled cannabinoid CB_1_ receptors^43^. Further, Chelerythrine induces production of reactive oxygen species (ROS)^44,45^ and participates in reversible complexation with cysteine^46^. Thus, chelerythrine may be involved in oxidative modulation of cysteines located in the RRM regions of TDP-43 as well. In addition, a close chelerythrine analogue sanguinarine with similar pharmacological properties has been identified as a potential TDP-43 interaction modulator in our initial screen (see Table 1).

In conclusion, we established the NanoBit complementation reporter assay to measure TDP-43 self-interaction and found two TDP-43 constructs with N-terminal tag as the strongest interacting partners. A screen of pharmacologically active compounds from the LOPAC^®^1280 library identified auranofin, riluzole and chelerythrine as inhibitors of TDP-43 self-interaction without reduction of TDP-43 expression. Pharmacological properties of all three substances and preliminary findings for auranofin may point to the hypothesis that inhibition of TDP-43 self-interaction is due to oxidative modulation of cysteine residues in RRM domains of TDP-43.

## Methods

### Ethical statement

We confirm that all methods were carried out in accordance with relevant guidelines and regulations. No experiments using live vertebrates or experiments using human tissue samples were conducted.

### Construction of plasmids

NanoBit^®^ luciferase complementation assay for protein interactions, N-terminal large (LgBiT, pFN33) and small (SmBiT, pFN35), C-terminal LgBiT (pFC34) and SmBiT (pFC36) vectors, constitutive NanoLuc luciferase pNL1.1.TK[Nluc/TK] vector ( = Nluc vector), positive control vectors SmBiT-PRKACA and LgBiT-PRKAR2A and negative control vector SmBiT-Halotag were obtained from Promega (Madison, WI, USA). Flexi^®^ entry vector with TDP-43 ORF have been bought from Kazusa Collection (clone FHC01302, Promega, Madison, WI, USA). TDP-43 protein-coding region has been obtained from the Flexi® Vector by a SgfI/PmeI cut and ligated into N-terminal fusion vectors (pFN33, pFN35) digested with SgfI/PmeI and C-terminal fusion vectors (pFC34, pFC36) digested with SgfI/EcoICRI, so TDP-43 is fused in frame with large bit of the split luciferase (pFN33, pFC34) or the small bit (pFN35, pFC36). Ligated constructs were transformed into high-efficiency *E. coli* competent cells (C2987H) and plated on kanamycin LB plates. The plasmids were purified from single colonies by QIAprep^®^ Spin MiniPrep Kit (Qiagen, Hilden, Germany) and NucleoBond^®^ Xtra Midi EF Kit (Macherey-Nagel, Düren, Germany). Correct coding sequence has been controlled by sequencing with sequencing primers (Table 3).

### Cell culture and transfection

Mouse neuroblastoma Neuro2a (N2a) cells were maintained in Dulbecco’s modified Eagle’s minimum essential medium containing 8% fetal bovine serum. Transfection of the indicated constructs in N2a cells was performed in 10 cm dishes using the Lipofectamine® 2000 Transfection reagent (ThermoFisher Scientific, Schwerte, Germany) according to manufacturer’s recommendation. 500 µl of Opti-MEM^TM^ media and 5 µg of plasmid DNA (2.5 µg pFN33_TDP-43 and 2.5 µg pFN35_TDP-43) have been mixed, while a second mixture with 500 µl Opti-MEM^TM^ and 12.5 µl Lipofectamine® 2000 has been prepared. Both mixtures have been incubated for 20 min at room temperature. 5.2 ml of Opti-MEM^TM^ have been added for a 6.2 ml transfection mix in total per dish. For transfection in 96-well plates 20 µl of the transfection mix have been added to each well containing 30 µl Opti-MEM^TM^. Twenty-four hours after transfection, N2a cells were plated into 384-well plates for the luciferase assay.

### NanoBit Luciferase assay

The assay is based on complementation of a split luciferase NanoLuc by the interaction of the tested proteins. For evaluation of the best configuration of the luciferase and TDP-43 fusion proteins, N-terminal and C-terminal fusion have been tested. Plasmid pFN33_TDP-43 and pFN35_TDP-43 yielded the best signal to noise ratio and are both N-terminal fusion of the luciferase large bit (17.6 kDa) and small bit (11 amino acids) respectively. Fusion protein expression is governed by the relatively weak HSV-thymidine kinase promoter, which yields TDP-43 fusion protein levels lower than the endogenous TDP-43 protein in N2a cells (Supplemental Fig. 3), so that cellular processes such as translation and protein sorting may not be overstrained with unphysiological quantities of TDP-43, which could lead to artificial cell behaviour. As a positive control, the interaction of PKA regulatory and catalytical subunit was used, and as a negative control, the absence of interaction with the HaloTag protein was monitored. Quantification of protein self-interaction was measured in the vital N2a cells in 384-well plates by addition of the NanoLuc substrate furimazine (Furimazine stock solution #N1130 1:800 diluted in Optimem, Promega, Madison, WI, USA), 5 µl of which has been added to give a final volume of 50 µl in each well. In our experiments, furimazine provided a stable measurement window of 15–60 min after injection (Supplemental information and Supplemental Fig. 1). For intermediate washes of the plates and medium change, we used Tecan HydroSpeed^TM^ (TECAN Group Ltd., Maennedorf, Switzerland). The luminescence signal has been measured in a Mithras LB 940 Multimode Microplate Reader (Berthold Technologies, Bad Wildbad, Germany).

### Cellular viability assay using WST-8

WST-8 assay is similar to the MTT assay with the exception that WST-8 does not cross the cell membrane. In the presence of 1-methoxy phenazine methosulfate WST-8 is reduced extracellularly to a formazane dye due to plasma membrane electron transport by NADPH-dependent cellular oxidoreductases^47^. The measured absorbance at 450 nm is correlated to the number and viability of cells in the analyzed well. WST-8 (2-(2-methoxy-4-nitrophenyl)-3-(4-nitrophenyl)-5-(2,4-disulfophenyl)-2H-tetrazolium, monosodium salt (Manchester Organics, Runcorn, UK) was used at 5 mM in 150 mM NaCl and supplemented with 200 µM 1-Methoxy-PMS (1-Methoxy-5-methylphenazinium methylsulfate, Dojindo Molecular Technologies, Munich, Germany). 10 µl WST-8/1-mPMS mix has been added to 100 µl medium in 96-well plates or 5 µl to 50 µl medium in 384-well plates. The resulting soluble Formazan dye formed within 60 min depending on cell density and absorbance was read at 450 nm.

### Screening for TDP-43 interaction modifiers using LOPAC^®1280^ library of pharmacologically active compounds

To screen for compounds modulating TDP-43 self interaction, the LOPAC^®1280^ library (Sigma-Aldrich, Darmstadt, Germany) has been applied. LOPAC^®1280^ library is a collection of inhibitors, receptor ligands, pharma-developed tools, and approved drugs covering most signaling pathways and major drug target classes. N2a cells were transfected with a 1:1 mix of pFN33_TDP-43 and pFN35_TDP-43 plasmids in 10 cm culture dish. Cells were subcultured in 384-well plates 24 h after transfection. Twelve hours later medium in 384-well plates was replaced by 50 µl medium containing luciferase substrate furimazine (Optimem, 1% FBS, 10 mM HEPES, 1 × NEAA and 1:400 dilution of furimazine stock solution from Nano-Glo® Luciferase Assay System #N1130, Promega) obtaining a reference luminescence measurement for each well. Afterwards, medium has been exchanged with 47.5 µl Optimem, 1% FBS, 10 mM HEPES, 1 × NEAA and 2.5 µl of the different compounds from the LOPAC^®1280^ library (stock concentration 200 µM) have been distributed to each well (final DMSO concentration 0,1%) and incubated for 60 min at 25–30 °C, followed by addition of 5 µl diluted furimazine substrate (1:400) and a second luminescence reading. Afterwards, 6 µl WST-8/1-mPMS mix has been added, incubated for 60 min and plates have been measured at 450 nm wavelength in a Mithras LB 940 Multimode Microplate Reader (Berthold Technologies, Bad Wildbad, Germany).

### Dose-response verification experiments with selected candidate compounds

To verify compounds identified by screening of TDP-43 interaction with the LOPAC^®1280^ library, we selected 7 substances (Table 1: GABA, Auranofin, Oxotremorine sesquifumarate salt, Riluzole, Suramin sodium salt, Reactive Blue 2 and Chelerythrine) to establish a dose-response relationship. Similar to the screening experiment pFN33_TDP-43 and pFN35_TDP-43 transfected N2a cells have been plated in 384-well plates and treated with each substance at final concentrations of 1.6 µM, 4.0 µM, 10.0 µM, 25.0 µM and 62.5 µM in a sixfold determination. Cell viability has been monitored by WST-8/1-mPMS assay as described above. In order to avoid false positive results due to pharmacological effects on protein expression, TK-promoter activity and interference upon luciferase activity we obtained in parallel also the luminescence reading of N2a cells transfected with the full length constitutively active NanoLuc luciferase (pNL1.1.TK[Nluc/TK] vector), which have been treated with the same substances.

### Quantification of endogenous TDP-43 expression after pharmacological treatment

N2a cells transfected with the constitutively active NanoLuc luciferase, which have been subjected to the dose-response measurement of the selected substances, were fixed with 4% PFA, 0.1% glutaraldehyde immediately after the WST-8 assay. Cells were stained for TDP-43 after blocking with 1% BSA with anti-TDP-43 2H4 rat antibody 1:4000 (BioLegend, San Diego, CA, USA) and as secondary antibody goat anti-rat Alexa-488 1:2000 (A-11006, ThermoFisher Scientific, Schwerte, Germany) and nuclei were labeled with 1 µg/ml Hoechst 33342. Afterwards, we obtained microscopic images from each well of TDP-43 fluorescence labeling and Hoechst 33342 fluorescence by using ZEISS Celldiscoverer 7 (Carl Zeiss AG, Oberkochen, Germany), an automated platform for cell imaging with autocorrection objectives to avoid spheric aberrations. Images have been processed to calculate TDP-43 fluorescence intensity by using Fiji (is just ImageJ, National Institutes of Health, Bethesda, MD, USA). The nuclear TDP-43 fluorescence intensity has been calculated as integrated density per area referred to marked nuclei in the corresponding Hoechst 33342 staining of the same well with adjusted uniform threshold. Calculated TDP-43 fluorescence intensity under auranofin, chelerythrine and riluzole treatment has been normalized to TDP-43 fluorescence intensity in untreated wells.

### RealThiol AM Ester Glutathion Detection assay

N2a cells were treated with 1.6 µM, 4.0 µM, 10 µM, 25 µM and 62.5 µM Auranofin and 0.1% DMSO as a vehicle control for auranofin in OptiMEM for 60 min at 37 °C. Cells have been harvested with Trypsine/EDTA and resuspended in FCS-containing DMEM. RealThiol AM Ester Glutathion Detection detection probe^48^ (Kerafast, Boston, MA, USA) has been added to the cells (100 µM RealThiol Working solution in 100 µl OptiMEM with final concentarion 1 µM) and incubated for 15 min at 25 °C. Afterwards, cell suspension has been diluted in further 900 µl OptiMEM and measured in a flow cytometer (Guava Easycyte) in FITC channel (Supplemental Fig. 4).

### Western Blot

N2a cells have been transfected with pFN33_TDP-43 and pFN35_TDP-43 construct as described above and incubated for 24 h at 37 °C. Cells have been lysed with 500 µl Laemmli lysis buffer per 2 wells of a 12-well plate (Tris/HCl, pH 6.8, 62.5 mM, 10% glycerol, 2% SDS, 5% β-mercaptoethanol, 0.1 M DTT). Proteins were separated using a 12% SDS-PAGE and subsequently transferred to a polyvinylidene difluoride (PVDF) membrane (PolyScreen; PerkinElmer Life Sciences, Boston, MA). Membranes were washed once in TBS-T buffer (150 mM NaCl, 20 mM Tris/HCl pH 7.4, 0.05% Tween 20), blocked in TBS-T containing 1% BSA (w/v), and probed with rat anti-TDP-43 2H4 (1:2000, BioLegend, San Diego, CA, USA). Detection of bound primary antibody was performed with rabbit anti-rat antibody (1:10000, DAKO GmbH, Jena, Germany). Blots were developed with ECL reagents and imaged in ECL-Imager (DNR MF-ChemiBIS Image Analysis System 1.6, DNR Bio-Imaging Systems, Biostep, Germany).

### Sequential protein extraction of TDP-43

N2a cells have been cultured in 6 cm dishes in triplicates either untreated, treated with 0.1% DMSO as a vehicle control or 10 µM auranofin for 60 min at 37 °C. Before scraping cells off the dish and pelleting cells at 400 × g and 4 °C for 10 min cells were washed with cold PBS. Each pellet contained about 17 mg of cell mass for each condition. Soluble cytosolic proteins were extracted with 200 µl PBS + protease and phosphatase inhibitors (cOmpleteTM Protease Inhibitor Cocktail, PhosSTOP, Roche, Basel, Switzerland) assisted by ultrasonication using three 2-second bursts at 40% maximum output (Virsonic cell disrupter) followed by ultracentifugation at 100,000 × g at 4 °C for 30 min in Sorvall MTX150 using S140AT rotor (ThermoFisher Scientific, Schwerte, Germany). TDP-43 contained in the insoluble particulate protein fractions was sequentially solubilized by 150 µl 1% Triton X-100 in PBS buffer (Triton X-100 soluble fraction) during a 60 min incubation at 4 °C followed by ultracentrifugation at 100,000 × g at 4 °C for 30 min. Thereafter, pellets were incubated with 80 µl 8 M urea containing PBS buffer (urea-soluble fraction) overnight at room temperature followed by ultracentrifugation. The resulting final pellets were resuspended in 20 µl 70% formic acid and agitated for 2 h at room temperature. Formic acid supernatant was neutralized with 100 µl 1.5 M Tris/HCl pH 8.8. 70 µl of protein extracts from each sequential extraction step were supplemented with 30 µl concentrated Laemmli buffer and 15 µl fraction were separated using 4–15% gradient TGX SDS gels (BIO-RAD, Hercules, CA, USA) and subsequently transferred to a polyvinylidene difluoride (PVDF) transfer membrane (PolyScreen; PerkinElmer Life Sciences, Boston, MA). Membranes were washed once in TBS-T buffer (150 mM NaCl, 20 mM Tris/HCl pH 7.4, 0.05% Tween 20), blocked in TBS-T containing 1% BSA (w/v), and probed with rat anti-TDP-43 2H4 (1:2000, BioLegend, San Diego, CA, USA). Detection of bound primary antibody was performed with rabbit anti-rat antibody (1:10000, DAKO GmbH, Jena, Germany). Anti-Hsp90 AC88 (1:1000, Enzo Life Sciences, Loerrach, Germany), Anti-PDI 1D3 (1:2000, Enzo Life Sciences, Loerrach, Germany) and Anti-Lamin A/C 4C11 (1:1000, Cell Signaling Technology, Danvers, MA, USA) antibodies were used as housekeeping genes for the different cellular fractions. Blots were developed with ECL reagents and imaged in ECL-Imager (DNR MF-ChemiBIS Image Analysis System 1.6, DNR Bio-Imaging Systems, Biostep, Germany).

### Gel filtration analysis and ELISA

N2a cells have been transfected with pFN33_TDP-43 and pFN35_TDP-43 in 10 cm dishes either treated with 0.1% DMSO as a vehicle control or 10 µM auranofin for 60 min at 37 °C before collecting the cells. Cells were extracted with PBS + protease and phosphatase inhibitors (cOmplete^TM^ Protease Inhibitor Cocktail and PhosSTOP, Roche, Basel, Switzerland) assisted by ultasonication. Supernatant (200 µl) of a 10 min 10,000 xg centrifugation step was applied to a Superose 6 10/300 GL column and eluted at 0.2 ml/min on an ÄKTApurifier (GE Healthcare Life Sciences, Freiburg, Germany) collecting 500 µl fractions. 50 µl of each fraction supplemented with 50 µl 0.1 M carbonate buffer pH 9.6 were used to coat 96-well Nunc MaxiSorp microplates overnight. After washing and blocking with 0.5% cold fish gelatine, wells were probed with rat anti-TDP-43 2H4 (BioLegend, San Diego, CA, USA). In addition, we used anti-TIAR1 (#8509, New England Biolabs, Ipswich, MA, USA) as a stress granule protein marker. The detection was performed in single well per fraction with HRP-conjugated rabbit anti-rat antibody (1:10000, DAKO GmbH, Jena, Germany) and tetramethylbenzidine (AJ Roboscreen, Leipzig, Germany).

### Statistical analysis

Verification experiments are performed more than twice to confirm the reproducibility. The results were expressed as the means ± SD of the indicated number. Unless otherwise stated the statistical analyses were performed by using Mann-Whitney-U-test and the statistical program GraphPad Prism® 7.0 (GraphPad Software Inc., La Jolla, CA, USA). p < 0.05 was considered to be statistically significant.

## Electronic supplementary material

Supplemental Information
