# Supplementary material for: TDP-43 self-interaction is modulated by redox-active compounds Auranofin, Chelerythrine and Riluzole
Source: Sci Rep. 2018 Feb 2;8:2248. doi: 10.1038/s41598-018-20565-0 (PMC5797228; doi:10.1038/s41598-018-20565-0)
Supplement: Supplementary file 1 — Supplemental Information [file 41598_2018_20565_MOESM1_ESM.pdf]

Supplemental Information to the manuscript „TDP-43 self-interaction is modulated by redox-active compounds Auranofin, Chelerythrine and Riluzole“

Authors: Oberstadt, Moritz[1*]; Stieler, Jens[2]; Simpong, David Larbi[2]; Römuß, Ute[1]; Urban, Nicole[3]; Schaefer, Michael[3]; Arendt, Thomas[2]; and Holzer, Max[2]

[1]Department of Neurology, University of Leipzig, Liebigstraße 20, 04103 Leipzig, Germany

[2]Department for Molecular and Cellular Mechanisms of Neurodegeneration, Paul Flechsig Institute for Brain Research, University of Leipzig, Liebigstraße 19, 04103 Leipzig, Germany

[3]Rudolf-Boehm-Institute of Pharmacology and Toxicology, University of Leipzig, Härtelstraße 16-18, 04107 Leipzig, Germany

Establishment of NanoBit interaction assay – Kinetics of NanoLuc activity

For further functional assays we tested at first the stability of NanoLuc activity signal depending on applied DNA amount over time (Supplemental Figure 1). Therefore, we used 100 ng, 50 ng, 25 ng, 10 ng, 5 ng and 1 ng of NanoLuc Plasmid DNA for the experiment and measured NanoLuc signal after 8 different timepoints (timepoint (tp) 1 = 0 min, 2 = 313 sec, 3 = 802 sec, 4 = 1729 sec, 5 = 2563 sec, 6 = 3412 sec, 7 = 4452 sec, 8 = 5321 sec). NanoLuc activity decreased the most in the 100 ng sample over time, if measured in total amount (mean at tp 0: 1704560 activity, mean at tp 8: 284861 activity), while the decrease was lowest in total amounts in the 1 ng sample (mean at tp 0: 7948 activity, mean at tp 8: 1463 activity, Supplemental Figure 1A, B+C). However, if the data are considered as decrease in % compared tp 1, all different concentrations of applied DNA show a similar decrease in % over time, e.g. for 100 ng NanoLuc Plasmid DNA (tp 1: 100%, tp 2: 94%, tp 3: 72%, tp 4: 48%, tp 5: 42%, tp 6: 31%, tp 7: 22%, tp 8: 17%) and for 1 ng NanoLuc Plasmid DNA (tp 1: 100%, tp 2: 88%, tp 3: 69%, tp 4: 48%, tp 5: 40%, tp 6: 32%, tp 7: 23%, tp 8: 18%, Supplemental Figure 1D). These data indicate, that decrease of NanoLuc activity over time is not dependent on plasmid DNA amount, which shows an excellent reproducibility of the assay for different plasmid DNA amounts. Regarding the stability of NanoLuc signal, the assay showed a reduction of NanoLuc signal to 50% (of the signal at tp 0) at tp 4 after 1729 sec (= 28.82 min, Supplemental Figure 1C+D). This result meant to us, that it is necessary to assure an early measurement after applying the NanoBit substrate to receive the highest possible signal.

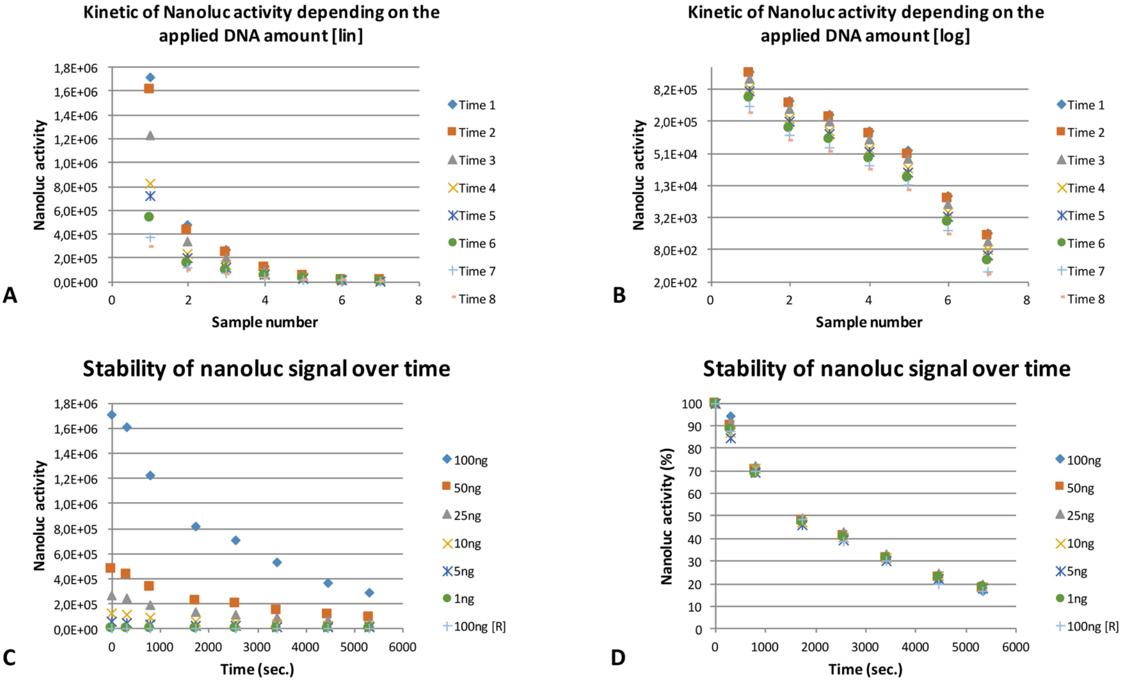

Supplemental Figure 1. Establishment of NanoLuc as a reference for NanoBit interaction assay
NanoLuc activity presented for different applied DNA amounts (sample 1=100 ng, sample 2=50 ng, sample 3=25 ng, sample 4=10 ng, sample 5=5 ng and sample 6=1 ng of NanoLuc plasmid DNA over time (timepoint (tp) 1 = 0 min, 2 = 313 sec, 3 = 802 sec, 4 = 1729 sec, 5 = 2563 sec, 6 = 3412 sec, 7 = 4452 sec, 8 = 5321 sec) in linear (A) and logarithmic diagram, with total NanoLuc activity (C) and NanoLuc activity in percentage of initial activity (D) over time.

Riluzole

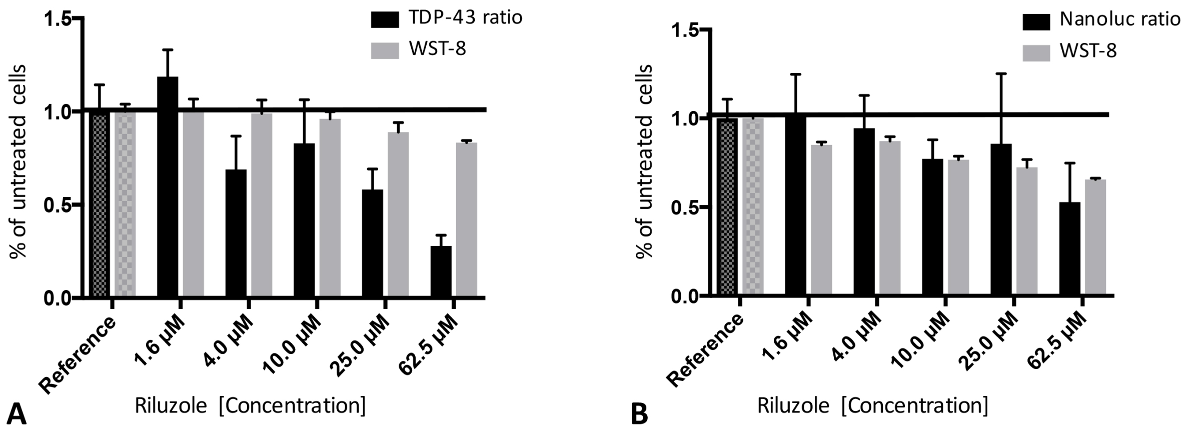

Auranofin

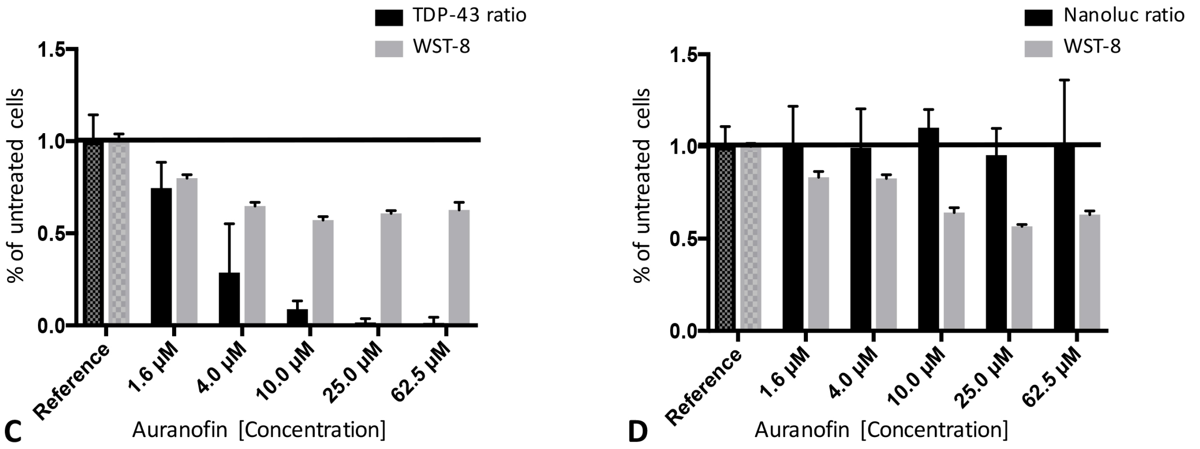

Chelerythrine

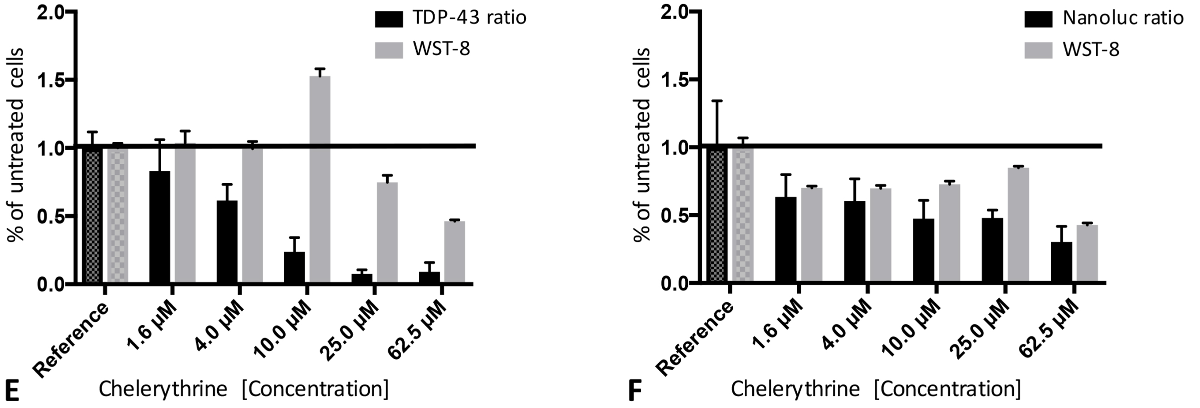

Supplemental Figure 2. Raw data TDP-43 self-interaction
Interaction of constructs pFN33_TDP-43 and pFN35_TDP-43 under treatment with compounds riluzole (A+B), auranofin (C+D) and chelerythrine (E+F) measured with NanoBit complementation reporter assay. Results described in % of untreated reference for each compound concentration (1.6 – 62.5 µM), in TDP-43 NanoBit luminescence and cell proliferation WST-8 assay for the same experiment (A, C+E) and as NanoLuc control NanoBit luminescence and cell proliferation WST-8 assay for the same experiment (B, D+F) for each treatment, Mean ± SD.

Supplemental figure 3.

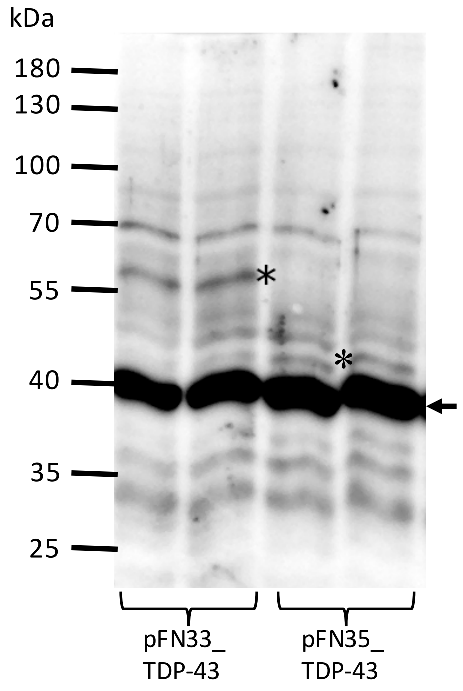

Supplemental figure 3. Western blot of endogenous and exogenous TDP-43 expression
Western blot of N2a cells transfected with TDP-43 constructs pFN33_TDP-43 and pFN35_TDP-43 after a 12% SDS-PAGE electrophoresis. The western blot has been stained with anti-TDP-43 2H4 antibody (1:2000, BioLegend, San Diego, CA, USA). Protein band for endogenous TDP43 (43 kDa) is marked by arrow, bands for transfected pFN33_TDP-43 LgBit (ca. 60 kDa) and pFN35_TDP-43 SmBit (ca. 44 kDa) are marked with stars in the figure.

Supplemental Figure 4

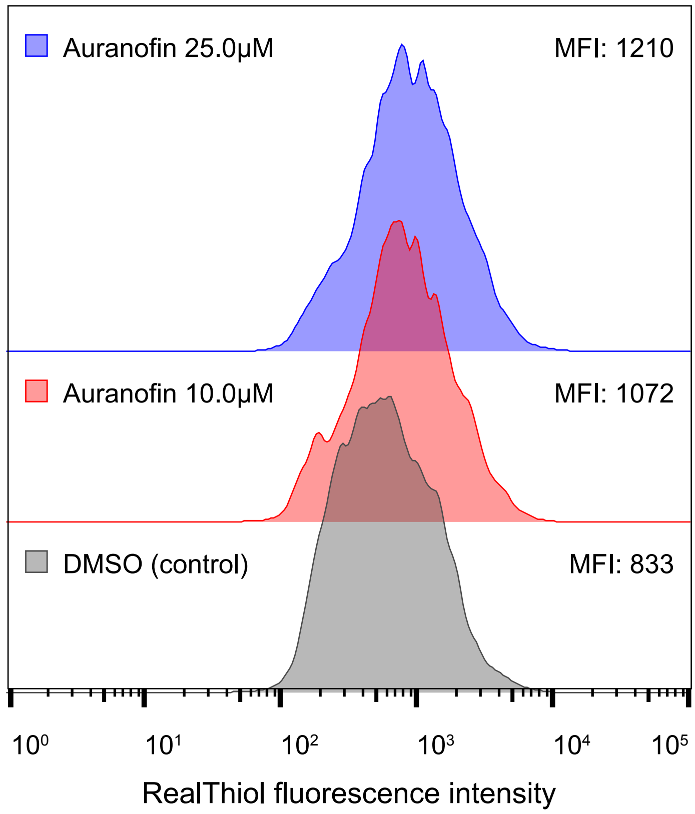

Effect of auranofin in RealThiol AM Ester Glutathione detection assay

N2a cells have been treated with 10 µM, 25 µM auranofin and DMSO (1:1000) as a vehicle control for auranofin diluted in OptiMEM for 60 min and RealThiol AM Ester Glutathione detection reagent (Kerafast, Boston, MA, USA) has been probed to the cells. RealThiol fluorescence intensity was acquired at the PE channel using a Guava easyCyte instrument (Merck Millipore, Darmstadt, Germany) and presented as stacked histogram.

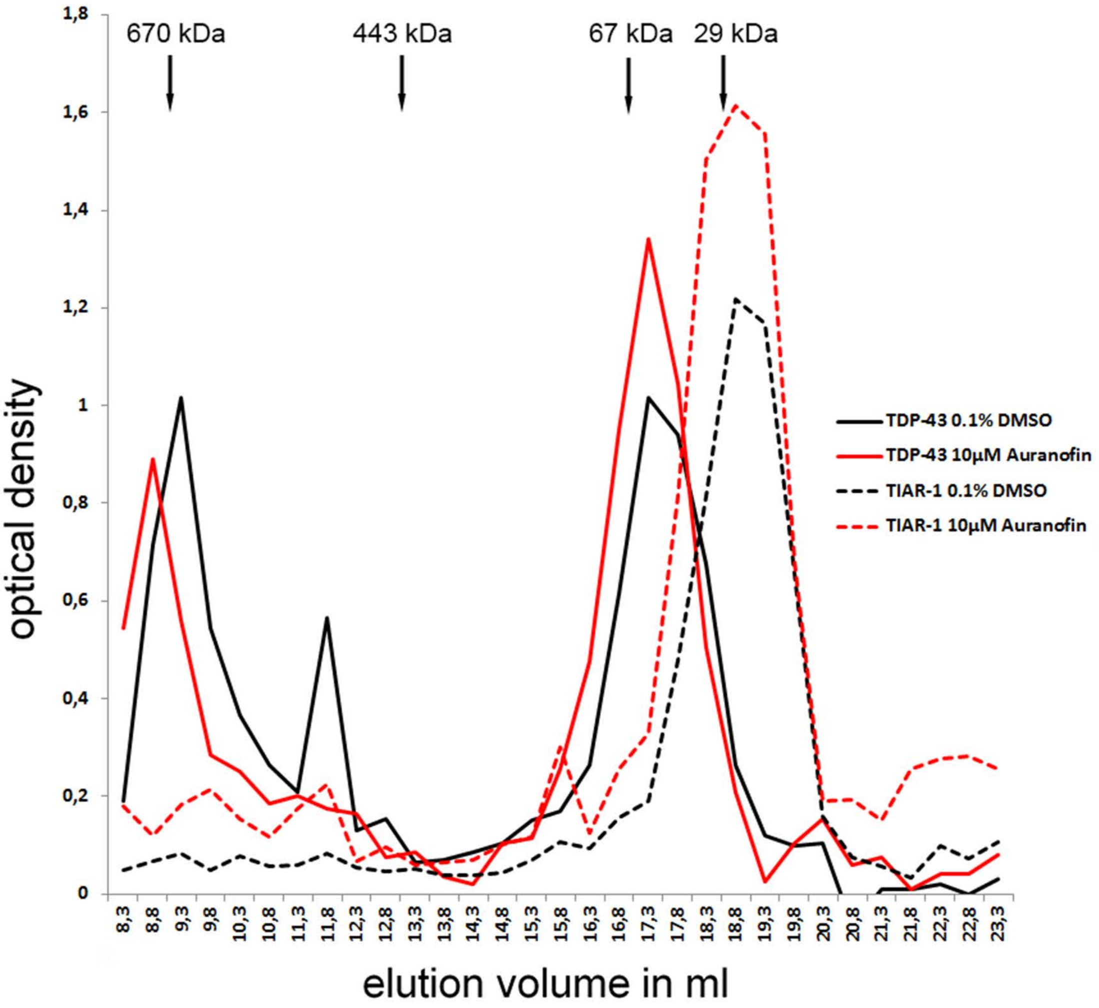

Supplemental Figure 5.

TDP-43 and TIAR1 immunoreactivity profile of soluble N2a cell extracts after size exclusion chromatography on superose 6 10/300 column. Quantification of TDP-43 and TIAR1 were perfomed using direct ELISA. TDP-43 proteins are eluted in two peak fractions with a similar size distribution in 0.1% DMSO vehicle control treated cells (black bold line) and 10µM auranofin treated cells (red bold line). There is no obvious change in molecular weight of TDP-43 fractions after auranofin treatment. Stress granule marker protein TIAR1 is not associated with TDP-43 and is eluted as a monomer (black dotted line) and unaffected by araunofin treatment (red dotted line). The elution profile of TDP-43 is similar to the size-separation performed on HeLa cell extracts by Kim et al. 2010.

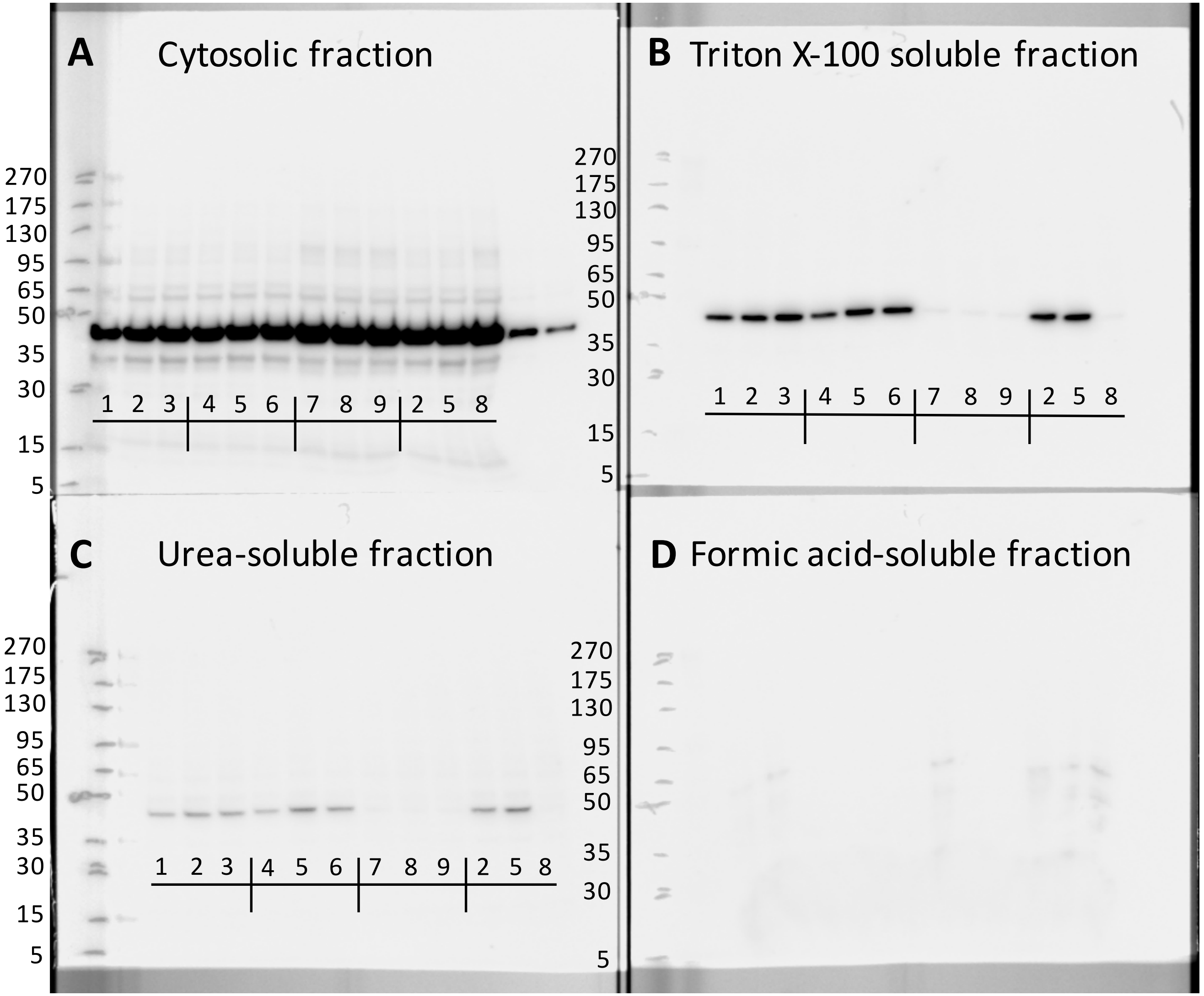

Supplemental figure 6.
Western blot raw data of sequential protein extraction of TDP-43 in equivalent order to figure 6. For further informations see legend of figure 6.
